# Supplementary material for: Collaborative intelligence in AI: Evaluating the performance of a council of AIs on the USMLE
Source: PLOS Digit Health. 2025 Oct 9;4(10):e0000787. doi: 10.1371/journal.pdig.0000787 (PMC12510544; doi:10.1371/journal.pdig.0000787)
Supplement: S2 File — (ZIP) [file pdig.0000787.s002.zip › S2 Data Raw data files containing unprocessed question inputs and LLM outputs including transcripts of deliberations/STEP3_Question-89-1_Type-MC-NJ.docx]

**Facilitator** **[Round 1]:** A 39-year-old woman, gravida 2, para 2, comes to the community-based health center because of a 6-month history of a copious, foul-smelling vaginal discharge. She also reports spotting that began 6 months ago and has progressed to heavy bleeding during the past 3 weeks. Medical history is significant for an abnormal Pap smear in her 20s; her most recent Pap smear was done 12 years ago during her second pregnancy, and she recalls the results as being normal. She takes no medications. The patient was married for 16 years; her husband died 4 years ago and had undergone vasectomy after the birth of their last child. The patient has not had any new sexual partners since her husband's death. BMI is 32 kg/m². Vital signs are temperature 37.2°C (99.0°F), pulse 90/min, respirations 14/min, and blood pressure 155/96 mm Hg. Speculum examination discloses normal vaginal mucosa. The cervix is friable with a 1-cm exophytic mass lateral to the cervical os at the nine o'clock position. Which of the following is the most critical factor in formulating a management plan for this patient? (A) Future fertility plans (B) Hypertension (C) Obesity (D) Patient age (E) Stage of disease

**AI_E:** RESTATING THE QUESTION: The scenario presents a 39-year-old woman with a history of abnormal vaginal discharge, spotting that now presents as heavy bleeding, and a concerning physical finding of a friable cervix with an exophytic mass. Notably, she has a history of an abnormal Pap smear in her 20s, but none recently. She is obese (BMI 32 kg/m²) and hypertensive. The question asks what would be the most essential factor to consider while evaluating and managing her condition. PLANNING: The factors listed for consideration are future fertility plans, hypertension, obesity, patient age, and stage of disease. While all these factors may play a role in patient outcomes, we need to consider the likelihood of more severe or imminent consequences. Given the history of persistent vaginal discharge and abnormal bleeding along with the physical finding of a friable cervix, a significant assumption here would be suspicion of a cervical pathology, possibly cervical cancer, which has stages that determine its management. CLINICAL REASONING: We first must evaluate the suspicious lesion by appropriate diagnostic testing, likely colposcopy and biopsy. Knowing the patient's age and past abnormal Pap smear result, and seeing a friable exophytic cervical mass, we might suspect cervical neoplasia, possibly cancer. While future fertility, obesity, and hypertension would be considered during treatment planning, the most imminent threat to the patient's health appears to be the possibility of advanced cervical cancer. If this suspicion is correct, it would be of most importance to determine the stage of the disease to inform the best treatment course. CONCLUDING: While all factors provided in the options are important, the most immediate threat to the woman's health depends on the severity (i.e., stage) of her potential cervical cancer. The stage of disease is critical because it will guide the urgency and aggressiveness of interventions and will significantly impact her prognosis. Making this determination should be the priority when planning her management. RESPONSE: Therefore, in the clinical context of this case, the most critical factor in formulating a management plan for this patient is (E) the stage of disease.

**AI_C:** RESTATING THE QUESTION: The matter at hand involves discerning the most vital determinant in creating a management plan for a 39-year-old woman exhibiting symptoms like copious, foul-smelling vaginal discharge, spotting leading to heavy bleeding, and a friable cervix with a 1-cm exophytic mass. She has a medical background of an abnormal Pap smear in her 20s, with the latest one being normal 12 years ago. She also presents with obesity and hypertension. The alternatives given pertain to future fertility plans, her hypertension, obesity, age, and the stage of her disease. PLANNING: The main assumptions to be taken into account includefactors which may significantly impact the patient's clinical outcome, these includes; her recent symptoms suggestive of a cervical pathology, history of an abnormal Pap smear, presence of a cervical mass, her hypertension, obesity, her age and fertility plans. Analyzing the assumptions, the central issue seems to be the cervical mass contributing to her symptoms. Considering this, the stage of the disease is a significant factor influencing management options. While factors like hypertension, obesity and age are important in a general health point of view, they do not directly impact the management of the present clinical issue - which appears to be a cervical pathology. CLINICAL REASONING: Cervical pathology is confirmed by symptoms like prolonged, malodorous discharge, spotting to heavy bleeding and finally presence of a cervical mass, which also has a history of abnormal Pap smear. Considering these symptoms, the possibility of cervical neoplasia cannot be ruled out. As with any malignancy, the extent (stage) of disease dictates both prognosis and appropriate treatment options. For instance, early stage disease might be managed with surgery or radiotherapy while later stages may necessitate combined modality treatment using chemotherapy, radiotherapy and/or surgery. CONCLUDING: While all factors presented in the options have their importance in a comprehensive clinical setting, the immediate issue is the potential cervical pathology exhibited by the patient. The stage or extent of the disease would most greatly influence what management approach is employed. Thus, the clinical priority in formulating the management plan would be determining the stage of the disease. RESPONSE: The most critical factor in formulating a management plan for this patient would be the stage of disease (E).

**AI_A:** (1) RESTATING THE QUESTION: The question asks which aspect is vital for deciding a patient's management plan, given her current medical history and clinical presentation. She is presenting with symptoms like abnormal vaginal discharge and a friable cervix with an exophytic mass, which hints towards a potentially serious gynecological pathology. It’s evident she had a medical history of an abnormal Pap smear and has not had one in the past 12 years, despite the recommended regular and frequent screening for early detection of cervical cancer. (2) PLANNING: Assumptions here would be that the patient's symptoms and examination findings might indicate cervical cancer. This could be due to the neglected follow-up after the abnormal Pap smear, possible HPV infection, and age. An alternate assumption would be that the symptoms and findings could also be an aggressive form of a benign condition, such as cervical polyp. Evaluating these assumptions, if this were early-stage cervical cancer, the choice between fertility-sparing management or more aggressive treatment could potentially make significant differences in her long-term prognosis and quality of life. However, if the diagnosis were a benign condition, this wouldn't present the same urgency. Therefore, it seems clinically most relevant to prioritize determining the severity and whether the patient's condition is benign or malignant. (3) CLINICAL REASONING: Foul-smelling vaginal discharge, spotting progressing to heavy bleeding, and a friable cervix with an exophytic mass are classical local symptoms that are very suggestive of cervical cancer. It is likely that her current symptoms are related to the progression of the disease due to the lack of regular and appropriate follow-ups since her abnormal Pap smear result. Therefore, to conclude on the best and the most appropriate management plan for this patient, we first need to establish the severity and stage of the disease. (4) CONCLUDING: Hence, by looking at the epidemiology and natural history of the disease, along with the clinical features presented by the patient, it seems highly likely that cervical cancer is on top of the differential diagnosis list. Given this, determining the stage of the disease would significantly impact the management plan, as treatment could vary from simple hysterectomy or even fertility-preserving management in early stages, to chemoradiation for advanced, invasive cancer. (5) RESPONSE: Therefore, the most critical factor in formulating a management plan for this patient would be the "Stage of disease" (Option E). It gives us a significant framework essential to decide the most appropriate treatment pathway, which could include surgery, radiation, or chemotherapy, depending on the severity.

**AI_D:** (1) RESTATING THE QUESTION: The question refers to a 39-year-old woman with a history of abnormal cervical cytology, who presents with a long-standing, foul-smelling vaginal discharge and an increase in vaginal bleeding. Upon examination, an exophytic mass has been found on her cervix. Of the factors given, we are asked to determine which would be most critical in developing a treatment strategy for her. (2) PLANNING: Firstly, the clinical presentation and findings, especially the exophytic cervical mass and history of an abnormal Pap smear, point towards the possibility of cervical cancer. The factors for consideration in management planning are her future fertility plans, her hypertension and obesity, her age, and the stage of her potential disease. Considering each in turn: (A) Fertility plans could affect decisions about radical surgery and radiotherapy that can cause infertility. (B) Hypertension could affect the choice of anesthesia and the possible risks of surgery, but likely would not be the most critical factor. (C) Obesity could also influence surgical risks, but again is unlikely to be the primary determinant of a management plan. (D) Age, at 39, can influence the type and intensity of treatments considered; younger patients may tolerate aggressive treatments better. Finally, (E) the stage of the disease could significantly impact the management plan, as more advanced stages may preclude certain treatment modalities. (3) CLINICAL REASONING: Decisions about a patient’s treatment plan often must account for individual circumstances and values, but the primary consideration should generally be treating the underlying medical problem effectively. Relevant important details in this case include the exophytic cervical mass and previous abnormal Pap smear, which increase the likelihood of a cervical malignant process. Given these findings, it's important to clinically prioritize the disease's stage, as it impacts both prognosis and the choice between possible interventions such as surgery, radiation, and chemotherapy. (4) CONCLUDING: Applying a step-by-step clinical thought process, confirmation of the disease's stage would be a crucial step. If the disease were in an early stage, local interventions like surgery or radiotherapy could be considered, while a more advanced stage might necessitate systemic therapy. Obesity, hypertension, fertility plans, and patient age, although important, would likely be secondary considerations to effective disease management. (5) RESPONSE: Thus, it can be said that the most critical factor in forming a management plan for this patient, who likely has cervical cancer, would be the stage of the disease (Option E).

**AI_B:** RESTATING THE QUESTION: A 39-year-old woman with a history of abnormal Pap smear and no recent screening, presents with foul-smelling vaginal discharge and heavy bleeding. Gynecological examination reveals an exophytic mass on her cervix. We are being asked which factor should be the most significant when shaping her medical management plan. The options presented are her future fertility plans, her hypertension, her obesity, her age, and the stage of her disease or illness. PLANNING: The assumptions imposed by her presentation and medical history include her potential risk of cervical cancer due to an abnormal Pap smear in her past, mitigated somewhat by a normal result twelve years ago, but complicated by a lack of recent follow-up screenings, symptoms suggestive of possible malignancy, and the finding of an exophytic cervical mass. Her stated lack of new sexual partners suggests a relatively low risk for sexually transmitted diseases. Other presumptions can be made based on her BMI and blood pressure, indicating obesity and hypertension, respectively. We could also assume something about her future fertility plans based on her age and her past gravidity and parity, but this isn't explicitly stated. When we examine these assumptions, we should logically focus on those that relate more directly to her presenting complaint and the associated cervical mass finding. Her obesity, for instance, while it can impact overall health, isn't directly related to her symptoms or examination findings. Similarly, her hypertension may not play a direct role in formulating the management plan for this particular patient. Her age is relevant to the degree that certain disease risks increase as a woman gets older, but it may not be as important as her disease's actual stage, which could significantly affect treatment options, prognoses, and overall management plan. Her future fertility plans could influence the type of treatment chosen if cancer is confirmed. While helpful in making decisions about the types of surgery or treatments that can be recommended, this is presupposing that a decision has been made about her disease stage. CLINICAL REASONING: If the woman's symptoms and physical findings are linked, which seems probable given their concurrent presentation, then the most clinically relevant factor for formulating her management plan should be the one most directly related to these symptoms and findings. Her age, while also important, may not be as relevant as actually determining the stage of the disease. Advanced-stage disease would possibly limit some treatment options and would carry a worse prognosis than early-stage disease. Similarly, if malignancy is confirmed, her future fertility plans would have a significant impact on the treatment to be chosen. However, this information isn't as urgent clinical priority as determining the nature and extent of the woman's disease. CONCLUDING: It is important to remember that without knowing the woman's personal wishes regarding future fertility, her preferences may still significantly impact her treatment choices if cervical cancer is confirmed. Yet, the crucial information that will shape the medical management plan for this patient is essentially the stage of her disease. Obstetric gynecologists typically categorize cervical cancer under four stages, with treatment options and prognosis sufficing significantly between stages. RESPONSE: Thus, the most critical factor in formulating a management plan for this patient is (E) the stage of disease. This information will provide a basis for prognosis, enabling us to discuss realistic treatment options with the patient with all facts at hand. It is also fundamental in shaping the immediate actions and interventions that should be taken for the patient's care.

**Facilitator** The consensus among the AI responses is that the most critical factor in formulating a management plan for the 39-year-old woman in question, who presents with symptoms such as abnormal vaginal discharge, heavy bleeding, and a friable cervix with an exophytic mass, is the stage of her disease (Option E). This conclusion is based on her medical history, which includes an abnormal Pap smear in her 20s and no recent screenings, and her current clinical presentation, which strongly suggests a potential cervical pathology, possibly cervical cancer. While other factors such as future fertility plans, hypertension, obesity, and patient age are important considerations in a comprehensive clinical setting, they are secondary to the immediate issue, which appears to be a cervical pathology. The stage of the disease significantly impacts the management plan, as it determines the prognosis and the choice between possible interventions such as surgery, radiation, and chemotherapy. Therefore, the clinical priority in formulating the management plan for this patient should be determining the stage of the disease.
